# Becoming ecological citizens: connecting people through performance art, food matter and practices

**DOI:** 10.1177/1474474015624243

**Published:** 2016-01-14

**Authors:** Emma Roe, Michael Buser

**Affiliations:** University of Southampton, UK; University of the West of England, UK

**Keywords:** arts-based, citizenship, food, food bank, matter, performance, practice, wellbeing

## Abstract

Engaging the interest of Western citizens in the complex food connections that shape theirs’ and others’ personal wellbeing around issues such as food security and access is challenging. This article is critical of the food marketplace as the site for informing consumer behaviour and argues instead for arts-based participatory activities to support the performance of ecological citizens in non-commercial spaces. Following the ongoing methodological and conceptual fascination with performance, matter and practice in cultural food studies, we outline what the ecological citizen, formed through food’s agentive potential, does and could do. This is an ecological citizen, defined not in its traditional relation to the state but rather to the world of humans and non-humans whose lives are materially interconnected through nourishment. The article draws on the theories of Berlant, Latour, Bennett and Massumi. Our methodology is a collaborative arts-led research project that explored and juxtaposed diverse food practices with artist Paul Hurley, researchers, community partners, volunteers and participants in Bristol, UK. It centred on a 10-day exhibition where visitors were exposed to a series of interactive explorations with and about food. Our experience leads us to outline two steps for enacting ecological citizenship. The first step is to facilitate sensory experiences that enable the agential qualities of foodstuffs to shape knowledge making. The second is to create a space where people can perform, or relate differently, in unusual manners to food. Through participating in the project and visiting the exhibition, people were invited to respond not only as ‘ethical consumers’ but also as ‘ecological citizens’. This participatory approach to research can contribute to understandings of human-world entanglements.

Stepping into the former shop from the busy high street in Bristol, UK, one meets the fragrance and aesthetic of green edible plants – aubergine, strawberry and tomato lining the entrance walls. Was this an indoor garden? Stepping further inside the plants stopped. Instead, clusters of small photos of food, plated-up, ready-to-eat or half eaten, were arranged on the walls. Emergency food aid recipients and volunteers took these photos. A variety of plates of food were on display: white rice, white toast, baked-beans, pasta, Weetabix, milk, Cheerios, Spam, pretty plates, place mats, green salads, sausages, chips, green beans and salmon. Some plates shared the same ingredients, yet the ‘look’ of the meal was very different. A number of photos shared a yellowy-brownie-beige aesthetic to the plates of food – it looked like a simple bean or tinned meat mixture was sitting on top of a round plate of rice or pasta. Plates of food that carried food of more varied shapes more often also carried a side-portion of something green. Some plates of food were positioned on tablecloths, others on a tabletop, a small side-table, another on a kitchen work surface, another balanced precariously on a radiator. Did visitors view these photos with disgust or salivation? On the floor of the exhibition room was a typical shopping basket of tinned and dry food given out by an emergency food aid provider. Moving in from the walls, the central activity sharply juxtaposed. Hands, fists and elbows working sticky, stretchy pizza dough or pummelling bread dough. Festivalgoers, food aid clients, passers-by, friends and family dropped in and out of this 10-day bread-baking extravaganza. On the back wall, there was a daily wordy titled ‘What Bristol ate yesterday’ – a daily overview of responses to a question posed to each visitor. In celebratory style on summer solstice night, food poetry was read, songs were sung, a dead chicken was plucked and people participated in transplanting seedlings to roomier pots (see Figure 1).

**Figure 1.**
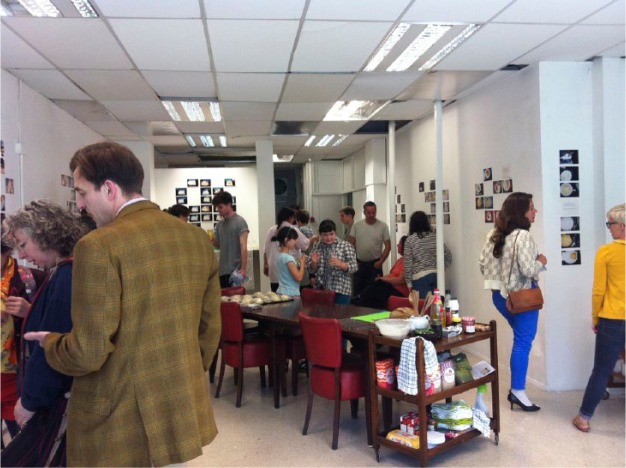
Visitors at the exhibition’s summer solstice event.

The exhibition was designed to allow for an open-ended interaction with performance artist Paul Hurley, artwork and various food-related materials and practices through both doing and witnessing. Participants were invited to discuss their food experiences (e.g. what they ate, how they accessed food) and to participate in various food-related activities. Sometimes visitors took home memories of conversations around food insecurity in their home city, but most often they took home the physical entity of a freshly baked loaf of bread and new cooking and baking skills through participating.

All told, over 900 visitors came into the space during *Big Green Week* and over 60 people baked. Within this art-space, visitors were confronted with the juxtaposition of food-related practices that carried the traces of food events taking place in different circumstances across the city. The intent was to create a space for sharing and exchange, different elements and different affects, and different opportunities to (dis)engage.

## Introduction

There is still a great deal of work to be done to explore how to engage, not necessarily to inform, Western citizens in the complex food connections that shape theirs’ and others’ wellbeing. Recent calls^1^ for developing understanding of the complex connections between agro-food provisioning and production systems, the environment and social justice have argued for these connections as a route to raising awareness about food security and access. How should one engage Western citizens who eat ‘Global Food’ (shorthand for a system of large scale, global food provisioning)^2^ to find out about, or to get a sense of, these complex connections? How can Western citizens recognise their own place within these complex connections and to equally grasp how it shapes personal health, the environment (including climate) and wider society? Finding answers to these questions is ongoing. One author responding to these concerns is Morgan;^3^ he has argued that for these connections to become politically meaningful will require a rethinking of the public spatialities of a politics of care, articulated through promoting the concept of ecological citizenship rather than relying solely on the actions of the ethical consumer in the private sphere. In this article, we try to put into practice Morgan’s aspiration, although admittedly using a different framing of ecological citizenship to the one he had in mind that is rich with insights from cultural geography.

In this work, there is the opportunity for cultural geographers of food and matter to engage existing understandings of how experiential knowledge shapes habitual food practices. This approach could inform pedagogic aims broader than influencing personal tastes for different food types on environmental or health grounds. Equally, cultural geography’s interest in thinking with a ‘more-than-human’ world,^4^ to map new ethical and political cartographies to connect bodies differently, disrupting bodily borders, need not only focus on the consumption and production of food but also on foods’ distribution and provisioning networks that arguably is as relevant to food security and access. How does one mobilise, generate and sustain a new politics of care around food that integrates practices and agentive edible matter that can shape the health of society, the sustainability of the environment and social justice? This is approached by describing a methodology that uses participative performance art for enacting ecological citizenship through sharing experiences of what people do with food – cooking, making a meal, shopping, growing and eating – in different circumstances. We draw on Latta’s invitation to think about nature and citizenship on very different terms, through taking seriously the agentic capacities of matter, and how this unsettles the categories of human and non-human. Our interest in food is different from the agentive materiality of water that featured in Latta’s study. We contend that this ontological approach to citizenship invites the materiality of food to become a political agent, which can support non-monetised, ecological ways of engaging and exchanging around food. Furthermore, it is argued that the creation of public and immersive spaces where people can think with and perform various food practices provides an opportunity to explore the complex material connections between humans and non-humans in food provisioning communities.

Our method for engaging people in the rich variety of food practices was through a co-produced, participatory arts-based research enquiry called Foodscapes. The opening paragraphs of the article describe the space where participation took place. Briefly, our project sought to unpack understandings and experiences of sustainability and resilience through direct engagement with individuals who suffer from food poverty and insecurity as well as the groups that work to address these challenges. These ideas were developed in various ways to create a performance art installation that received visitors for 10 days. The various strands of the project engaged audiences, research participants and community partners through a focus on sharing different practices of working with edible matter to make food, including shopping, baking, planting and preparing a meal. We explain and argue in this article how through both reflecting on and taking part in food practices in our performance space, people began to perform the role of ecological citizens. We propose that this finding can help stimulate other types of food-related activities, including participatory art practices that can support the development of becoming ecological citizens. In this manner, acknowledging Guthman’s^5^ critique of race-related ideologies that drive the quality food agenda within many alternative food initiatives, we focus not on food quality but on food access and insecurity.

The article begins by discussing the use of participatory arts practice as a vehicle for bringing care-receivers and caregivers (emergency food donators, volunteers) into a space where different food practices can be enacted. Then, the agro-food studies literature concerned with food practices, food as a performance medium and the politics and ethics of agentive edible matter is discussed in terms related to the spatialities of agro-food production, processing and consumption. These literatures are discussed to explain the central argument that a performative approach when applied to the process of knowledge making and role-formation enacts an ecological citizen in our novel terms. Within the local food initiative literature arguments exist, for example by Morgan^6^, for the importance of kindling ecological citizenship in publics and the shortcomings of appealing to the ethical consumer. We contrast our concept of becoming ecological citizen with Morgan’s enrolment of the ecological citizen within democratic state processes, to instead appeal to becoming an ecological citizen, untethered from a relationship to the state, always instead alongside, responding with, and to, the agentive materiality of nature. Thus the argument follows a Latourian^7^ political manoeuvre that locates the political as generated in the making of quasi-objects, hybrids, material objects, which trouble the modern boundary between nature and society, human and non-human, ecological and non-ecological. The politics of food are the politics of nature as food politics cannot avoid the handling of non-human animal and plant bodies, but also must grapple at some level with the corporeality of human and non-human bodies being eaten and eating, an ecological process of matter exchange across bodily borders. After outlining the theoretical background to how we define ecological citizenship in relation to food studies, we move onto apply this thinking to our interpretation of a 10-day performance art installation/exhibition. In the methodology, we describe how we worked with two community-oriented local food initiatives in Bristol, UK, to develop components of the art installation. In our reflections on what went on here, we draw on interviews and survey results to convey the experience of being in this space and propose participants engaged in becoming ecological citizens. We then outline two steps for arranging the performative space to support visitors to enact the role of ecological citizen. The first is facilitating sensory experiences that enable the agential qualities of foodstuffs to shape knowledge making. The second is creating a space where people can perform or relate differently to food. Finally, we argue adopting this approach to becoming ecological citizen facilitates fostering care for others’ food insecurities.

## Participatory art and creative arts practice as research

Popularised during the 1960s and 1970s, arts-led socially engaged practice (often known as participatory art) has been used to ‘democratise’ arts practice by challenging traditional (sometimes didactic) relationships between artist and audience and distributing authorship away from a central figure (i.e. the artist). Participatory art commonly seeks to shift the role of the audience away from observer or spectator and towards an active involvement as producers of works of art and performance. As Claire Bishop^8^ points out, participatory art challenges conventional capitalistic modes of artistic production and consumption by troubling both the commodity form of art and the role and exclusivity of the artist. Commonly, these participatory art practices endeavour to renew so-called fragmented bonds by forging new collectivities through arts-based social action. In the United Kingdom, during the late 1990s, the positive impact of participation in the arts was taken up by the New Labour government drawing on a report by Francois Matarasso^9^ who set the range of possible benefits of such practices – including reduced isolation, increased employability and so on. These arguments influenced the subsequent surge of participatory and socially engaged art practices where process, collaboration and engagement have since come to the fore. Much of this work is action-oriented and consistent with participatory processes in the social sciences where work is co-produced by researchers and participants in order to produce real-world, practical solutions to pressing concerns.^10^ This includes a shifted authorial emphasis where the artist is ‘less conceived as an individual producer of discrete objects [but more] as a collaborator and producer of situations’.^11^ Within the arts, projects have explored the social benefits of making connections between food and creativity by bringing people together in collaborative food provisioning endeavours.^12^

Coupled with the interest in participatory work, there also is a move towards an understanding of arts practice as a mode of research enquiry – ‘as the production of knowledge’ where ‘knowledge is derived from doing and from the senses’.^13^ This thinking develops from close examination of artistic practices with materials and how the materials afford knowledge production through practical, sensory engagement with them. Estelle Barrett claims materialist practices provide an alternative logic to traditional scholarship that has conceived materials as inert and the creative process as uni-directional from artist to ‘canvas’. As art theorist Bolt puts it,

[T]he materials are not just passive objects to be used instrumentally by the artist, but rather, the materials and processes of production have their own intelligence that come into play in interaction with the artist’s creative intelligence.^14^

Foodstuffs – flour, dough, olive oil, salt, water, yeast – and food equipment (bowls, wooden boards, spoons) are the materials of the everyday, and yet through artistic practice, interacting with them intensively and creatively possibilities emerge for new ways of knowing to emerge.^15^ This approach to participatory arts shares the same ontological flexibility to knowledge creation found also in food studies that study the agentive politics of matter (discussed later). Important is the premise that humans create and know-about something not through isolated, disengaged pondering, but through active engagement with a lively world. Therefore, doing research or exploring what we can understand in the world, for academics, artists and indeed anyone, is through interacting with materials. By building on this way to understand lived engagement with a material world, we turn to consider what this might offer a politics of care practices. The suggestion we make is that this approach can engage people in the process of becoming ecological citizens, through different active relations with foodstuff.

Rather than focusing on the evaluative mind of a subject who through experience comes to understand consciously what it is to care, we work with an embodied, human subject who is affectively enrolled through life in what Berlant describes as a ‘pedagogy of emotion’.^16^ Berlant describes how we learn to feel through being affected. Through this process, an affective aesthetics of what objects (including people) make one feel fearful, happy or worried becomes established. Thus, feelings emerge historically in the life of an individual about who to care for and how to do it. For us, participatory arts’ practice offers experiences that can play a part in an aesthetic pedagogy of emotion, which in turn informs the becoming of ecological citizens. This may happen through making material aesthetic connections through practices of doing. This is opposed to developing a conscious regard for others or an intentional approach to enrol as citizen. This shift from enrolment to becoming citizen is made through interpreting the embodied more-than-human subject as always entangling with other human and non-human materialities through practices. It is through participatory art practice that care can be located when a particular affective aesthetic affords the forging of the carer and cared-for. In the next section, we develop these ideas further through providing some background to how this approach has developed in agro-food studies and how the politics of matter creates space for the becoming ecological citizen.

## Politics and ethics of agentive edible matter

Over the last couple of decades, agro-food studies have increasingly engaged with the materiality of foodstuff and food-related practices. Much of this work is characterised by rich ethnographic detail of exactly how things become edible food, foregrounding the choreography between matter and practice that creates an ongoing flexibility of meanings to different (in)edible materialities. This engagement with the choreography of food matter and practices demonstrates the influence of performance studies where food is staged as both performative (agentive) and a medium for performance, illustrated in the work of Kirshenblatt-Gimblett^17^ and Bobby Baker.^18,19^ This discipline creatively explores different material connections and meanings associated with food. It also reminds us that the social and cultural practices that surround edible matter is more than a set of nutritional properties or the combination of ingredients in a recipe. For example, to be successful in creating a meal or growing something edible, one often needs additional expertise or prior experience. The skills for knowing the duration to knead dough for, to stir a mixture effectively, to make a sauce, to transplant young plants carefully or to become sensitive to signals about smell, colour, texture and taste are all crucial to the highly variable processes of growing, processing and cooking different foodstuffs. Engagement in these processes and practices can be transformative in terms of both what and how edible matter is known.

Typically, the most common connection made to achieving social justice for other food ‘communities’ is to growers, producers and labourers through commercially driven fair-trade initiatives.^20^ Equally, the ethical concern for food production standards is familiar territory for consumers, successfully turned into a niche in the food product market (e.g. organic food-labelling and animal welfare–friendlier foodstuffs). Allen^21^ acknowledges how social justice is not achievable through alternative agricultural and food systems such as these examples above. Instead, she argues for academics to challenge the categories of inquiry and how the problem is being defined, a clear intent of this article. For example, there are other cartographies of material connections that can be mapped to connect communities around food. Instead of looking upstream and downstream within the supply chain for material connections, one can look across to those people who despite eating at a different dining table, a material connection can be traced with them through what is eaten, although very rarely is this material connection precisely known. Unknown material connections can be present in food donated anonymously from one to another, or between pieces of meat sitting anonymously on different plates yet carved off from the same animal carcass in the abattoir, or the anonymity of preparing meals with similar ingredients bought from a high street supermarket, but in quite different personal circumstances. There is no commercial interest in marketing or drawing attention to these connections through selling food with this kind of story.^22^ This is no ethic of how food is being produced at stake here, arguably instead this relates more to the material ethics that connect between bodies through food’s distribution and provisioning networks.

Through a combination of artistic practice and treating food as both performative and a medium of performance, it is proposed that the material ethics within food distribution and provisioning networks could be made more visible. Importantly, this could facilitate greater consideration towards food access and food (in)security. Bennett describes edible material as ‘agent inside and alongside intention-forming, morality-(dis)obeying, language-using, reflexivity-wielding, culture-making human beings’.^23^ Thus, making matter connections more visible can happen through human practices and non-human matter assembling in a particular way to create an event that shapes human intention, morality, reflections or cultural practices, but importantly it is not read as the outcome of intention, moral reflection or established cultural practice. In other words, in certain scenarios, edible matter can become an agent in bringing attention and concern to its activity. What interests us is how an experience can be created that enables edible matter to be heard, felt, sensed and thus to make connections that are less often realised and have not become a recognisable ‘matter of concern’^24^ to people. The proposition is that during edible matters’ intra-activity with humans, previously invisible connections between peoples and matters could be made visible, becoming in the process a ‘matter of concern’.

## Ecological citizenship and food

Without a matter of concern, Latour^25^ argues, the becoming citizen is unable to engage with the politics of the ecological. Therefore, as Latta writes, ‘Locating citizenship in relation to “matters of concern” requires an ontological flexibility to the numerous ways that peoples and nature *become political*’.^26^ Within encounters between agentive edible matter and becoming citizen, matters of concern may emerge related to food security, food poverty, food gluttony, food elitism, unhealthy food and environmental damage from food production, to name some possibilities. It could be a personal anxiety about what they ate yesterday – or a realisation of the plight of others in food circumstances different from their own. This ontological flexibility facilitates people becoming political, by drawing people through food practices and different food materials (in the widest sense) to make who-knows-what form of ‘matter of concern’ and thereby align themselves with ecological citizenship. As Latta writes,

Through its performances and interjections, matter becomes more than simply a set of properties to which human actors respond, and rather another embodiment of the insurgent qualities associated with the political becoming of citizenship.^27^

This approach to the political becoming of citizenship is unexplored in relation to food. However, within the politics of food literature, there is interest in the potential of the concept of ecological citizenship and global citizenship^28^ to raise awareness of food connections. Morgan^29^ argues that for food connections to become politically meaningful will require rethinking the public spatialities of a politics of care, articulated through the concept of ecological citizenship not only the actions of the ethical consumer. Following ethnographic analysis of learning about global citizenship through fair-trade food, Pykett et al. describe how the institutionalisation of citizenship education in schools ‘aims to govern subjects through cultural practices reflecting a diverse set of interests, commitments, and comportments’,^30^ but that their study indicates that these pedagogic practices struggle to achieve intended ‘subject-effects’ or aims. Interestingly, because of the fear that these pedagogic interventions interfere with the free subject, these pedagogies actively invoke the norm of autonomy and the discourse of ‘choice’ in relation to consumption practices.^31^

It is a different conception to ecological citizenship proposed here than what Morgan, developing Dobson’s^32^ original thinking, envisages or what Pykett et al. describe. Morgan’s position could be described as the enrolling of the ecological citizen through the infrastructure of the democratic state, whereas we propose the becoming of the ecological citizen through the embodied, more-than-human learning to care, to be affected through intra-actions, in diverse forms with humans and non-humans. There are three key distinctions. First, unlike Dobson’s initial conception of ecological citizenship that Morgan discusses in relation to food, the ‘becoming ecological citizen’ is not defined by embodying a political will or hope that it is possible to address food access and sustainability issues across the globe. It is not advocating for a politics associated with democratic action as the route to address social justice. Second, it is argued by Dobson that ecological citizens care because they are motivated by social and environmental justice, and indeed this leads Morgan to argue for a rethinking of the public spatialities of care:

We care for others because this is what being sustainable means in an ecologically interdependent world. The fact that some citizens may be motivated less by disinterested notions of social justice and more by enlightened self-interest neither diminishes nor invalidates the basic argument.^33^

The emphasis on how care for and about others or themselves can follow seamlessly on from understanding ecological interdependence is ignoring the affective process through which emotions, like care and compassion, are learnt. Who or what to care for is not learnt through understanding cold abstract concepts like ‘sustainability’. Similarly, Pykett et al.’s study indicates that where role-playing games are used to engage children in embodying the experience of receiving low wages for picking coffee beans, ultimately the pedagogic appeal is to cultivating conscious, rational reflective subjects. Finally, third, Morgan emphasises that those in need of being cared for need to participate with renewed commitment to democratic processes. Whereas those who need caring for can participate equally in becoming ecological citizens alongside carer, or a fellow cared-for, by sharing with others their personal rich and diverse experiences of eating, preparing and shopping with agentive foodstuffs. Those in receipt of care do not have to vote or approach a member of parliament (MP), along the lines of formal democratic processes, but can instead share their own food skills and knowledge.

In summary, the three literatures – participatory art practice, the politics and ethics of agentive matter, and ecological citizenship and food – lead to formulating the following two questions. How does the performance arts-space offer diverse experiences, skills and practices that support becoming ecological citizen? How can performance art engage people to sense food connections among eaters and eaten in the distribution and provisioning of food?

## Methodology: co-producing work with non-academic and non-human participants

Our collaborative project was between academics, Knowle West Media Centre (a local community media organisation) and two local food initiatives – The Matthew Tree Project (TMTP) and the Edible Landscape Movement (ELM) – in the city of Bristol, UK. In varying ways, all of our non-academic partner organisations have been working to address ‘hidden hunger’^34^ and food poverty. The TMTP is a volunteer-run free-food provisioning service that offers local people-in-need a mock shopping experience of food donated by local supermarket shoppers across the major food groups; they call this a Foodstore, rather than a food bank. The organisation offers food as a way to then help people with a range of social welfare problems such as debt, seeking asylum, benefit-delays, addiction and family break-up. Many would describe it as a form of emergency food aid provision, along similar lines, if not identical, to a food bank.

The ELM, supported by Knowle West Media Centre (KWMC) is a community food growing and provisioning project that supplies fresh vegetables, fruit and baked goods to a community living in an area that meets the criteria for a ‘food desert’.^35^ Thus, even within regions where food supply is plentiful, there can be ‘hidden hunger’ in the form of food deserts in the middle of cities where local people have no access to fresh foods, where small grocers have been forced out of business and consumers must drive to distant supermarkets to purchase their foods.^36^ Those without access to a car or whose mobility is otherwise limited, either through physical or financial limitation, are increasingly vulnerable in such areas.

In various ways, these organisations operate to more or lesser degree outside the corporate food mega-complex. Their activities respond to a marked increase in the United Kingdom, of the numbers of people struggling to keep above the food poverty line. In 2014, the Trussell Trust reported that over 900,000 people were fed by food banks in the United Kingdom, up 163 per cent increase from the previous year.^37^ This increase in need has been accompanied by a dramatic expansion of emergency food aid service providers.^38^ Emergency food aid is usually a last resort for people who do not have enough money for food after other expenses.

What is critical about the success of emergency food aid and community-growing projects is the role of local people as volunteers, food donators or food growers to maintain the availability of emergency food aid. Meanwhile, retailers and corporate interests may view their donation of surplus food as good citizenship in that it assists the needy, prevents waste and reduces dumping and disposal costs, but it also means that food banks become entwined with corporate needs and a second-tier food system that depends upon the corporate global food sector becomes entrenched. Furthermore, some argue that such practices obscure more structural issues related to food poverty and undermine the State’s obligation to address food poverty and nutritional health and wellbeing.^39,40^ Interestingly, these food initiatives are not defined by a sole focus on locally produced food, but rather focus on locally organised provisioning and distribution systems. The practices associated with these food provisioning systems are forged on caring for those who are vulnerable to food insecurity, rather than addressing concerns about supporting a local food economy and reducing the distance food travels. In effect, they are enabling a different style of engagement at a local level around food, to that commonly associated with the retailing of locally produced food.

Foodscapes sought to explore ideas about sustainable futures in concert with individuals who suffer from food insecurity. We examined food practices (e.g. accessing, eating, cooking and sharing food) and looked at the role organisations such as TMTP and ELM played in addressing these challenges. The project began as an exploration without defined research questions, detailed methods or expected outputs. In order to refine our contextual understanding, we held a series of group meetings and focus group workshops, volunteered with TMTP and visited projects run by our project partners ELM and KWMC. The artist Paul Hurley was employed by KWMC to deliver an intervention on producing and accessing food, and to develop creative ideas informed by exchange with participants, the Foodscapes team and other stakeholders. The two primary researchers and the artist conducted in total 150 hours of participant observation with community partners at their sites as well as during subsequent arts activities. Prior to the development of the installation, an evening of focused-discussions around food experiences, knowledge, anxieties and finally ideas about what they would like Foodscapes to produce was held with community partners and volunteers. This evening along with the participant observation and sustained and regular contact between the project partners created an atmosphere that the artist Paul Hurley described in an email^41^ as

organically collaborative, in generating ideas and ways of working – I don’t know whose were what now, and for certain couldn’t have got here alone. I guess volunteering has not only provided a bedrock of understanding first hand the situation, but introduced inescapable affective experiences of empathy, compassion, humility and (in)justice. But also a sense that our actions have been part of the solution, if only in the very short term.

In addition, surveys were conducted with 70 visitors to the arts installation, and a further 20 follow-up phone interviews with participants, and two group interviews with core members of the Foodscapes team.

During group discussions, we decided to focus our arts and creative work on *Big Green Week* (a 10-day national sustainability festival held in Bristol) to magnify the project’s profile and draw attention to the issues of food security. All community partners were actively involved in what would happen at the exhibition. Still at this stage, there was uncertainty about the work that was being made to exhibit. It is only latterly that reflection by the research team has enabled us to work out what went on. Indeed, for example, as we discuss in detail below, while the research involved the active involvement of community groups to support the direction and activity of our work, we also recognised that the foodstuffs themselves were as important in shaping the scope of our activities. These were not just passive objects but agentive materials generating ideas and practices in the events that unfolded.

**Figure 2.**
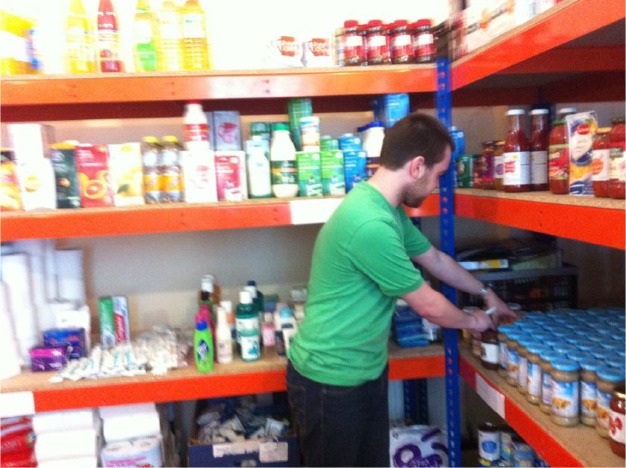
A volunteer with TMTP stocking shelves in the foodstore.

As we were dealing with people in precarious life situations, we sought non-obtrusive but engaging ways of thinking about the daily experience of food insecurity (including how people accessed food, the types of decisions they made about diet) and the condition of food poverty with volunteers and clients. At TMTP, clients in need are not handed a food parcel. Rather, following an interview with staff, they perform a shopping experience, selecting their items from shelves in the foodstore stocked with food donated to the charity. We were impressed by this purposeful and constructed experience of ‘shopping’, which brought elements of choice and performativity into the practices of receiving emergency food aid (see Figure 2). However, we were also interested to know how clients cooked, supplemented and created meals with these items once they left the foodstore.

Thus, following a few weeks of working as volunteers and speaking with people accessing food through TMTP, our group decided that photo-voice methods would be an ideal mechanism to share food experiences outside of the foodstore environment. Photo-voice is a participatory research approach in which people use video and/or photo images to capture aspects of their environment and experiences for sharing with others.^42^ Clients and volunteers were provided with disposable cameras. They were invited to take pictures each mealtime of what they ate over a period of a week. The following week, when returning the cameras, they were invited to discuss how they found using them.

In the next section, we analyse experiences of the event-space, described in the opening paragraphs of the article, in further detail. We outline two steps for supporting people to perform the role of ecological citizens via food connections across diverse communities and provisioning practices. We argue food is an effective vehicle for approaching how people can be encouraged to perform the role of ecological citizenship. Within this context, we argue it is important not only to have sensory experiences with materials to encourage the exchange of skills and practices but also to foster a space that is experienced as a juxtaposition of various food materials and practices from diverse people; together these encourage the performance of ecological citizenship.

## Discussion

### Becoming ecological citizens: Step 1 – facilitating sensory experiences that enable the agential qualities of foodstuffs to shape knowledge making

Mike Carolan^43^ has argued for the potential of tacit embodied food practices for fostering new sensual experiences that involve feeling, smelling and eating food differently as part of a community-situated food revolution against Global Food. We share his interest in developing performative political engagement through sensory engagement with foodstuffs. For Carolan, this is a politics that revolts against what he calls Global Food – food produced by commercialised global supply chains:

Many people go their entire lives without stepping on a farm or garden, knowing the tilth of rich, organic soil between their fingers. [. . . ]. What I mean is that food *itself* is becoming transparent.^44^

While we support Carolan’s concerns, bringing people in touch with how food is produced was only one dimension to this project, and it would be fair to say we were not revolting against Global Food but rather developing a deeper awareness for the ‘ecological’ complexity of different food practices that connect and disconnect peoples and the food distribution and provisioning environment broadly conceived. For us, embodied food practices were politicised not only through the act of doing them but also through the juxtaposing of different food-related experiences. A variety of sensory food practices were presented or evoked in the space, and people were left to engage sensorially, reflect or ignore what and how food was grown, cooked with, made into a meal and eaten by different people. This tactic was inflected by an approach to contemporary food policy^45^ that seeks to integrate environment, health and society concerns around how food policy and politics are taken up in society. In contrast to Carolan’s work, we argue that the breadth of embodied engagement we sought fostered the performance of ecological citizenship, as something different to a revolt against a particular type of supply chain, but rather for promoting understanding of the interconnectivities between diverse, yet shared food practices.

The exhibition brought together experiences, materiality and aesthetics of our diverse project partners (the food bank, the polytunnel, the bakery, the kitchen) into a performative and multisensory space. This offered visitors a direct bodily engagement – kneading bread, smelling tomato plants, experiencing the weight and food types within a shopping basket of a week’s emergency food aid: these practices we came across through the experiences of getting to know our project partners’. As bread was a central feature of our exhibition, the qualities and characteristics of ingredients such as flour, water and yeast afforded particular experiences. For example, involvement in bread baking meant sticky hands, a slow repetitive pace of kneading, attention to the texture of dough and a more precise awareness of time and intensities such as heat and air:

It amazes me you can just mix these two dry ingredients, or three dry ingredients and some water and it’s like a sticky mess, and then just kneading action with it and then it changes, and then you just leave it and it changes again, and then put it in the oven and it changes again into bread.^46^

**Figure 3.**
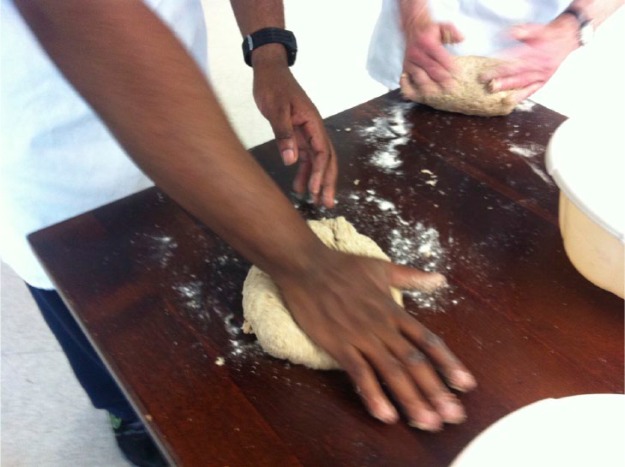
Exhibition visitors kneading dough and baking bread.

These experiences are similar to what de Certeau^47^ has discussed in terms of the mystery, fascination and appreciation of the sourdough ball – certainly, the practice of baking enchanted many of our visitors who got caught up in the slowness of the processes and so time opened up for taking part in dialogue, communication and exchange – hands kneading bread, slowly, rhythmically – sharing ingredients, sharing stories, sharing knowledge (see Figure 3). In these moments, we forged new types of connections that shaped physically the bread we made, the knowledge imparted and shared and the stories offered. In follow-on interviews with two visitors, these reflections were captured:

What a welcoming open space it was, as an art gallery and as something doing more than art . . . The space was very engaging. The bread making was amazing. You could participate or just watch.^48^

It was just an empty shop, but it was quite welcoming, with the smell of baking and plants up the wall.^49^

These and similar comments underscore the sense of the unexpected sensory experience the space offered and how particular food materials co-produced a welcoming and engaging space.

### Becoming ecological citizens: Step 2 – creating a space where people can perform, or relate differently, in unusual manners to food

By welcoming our partners and the general public to a lively arts and festival space, participants were encouraged to shed consumerist ethics in favour of becoming an ecological citizen. This was achieved through inviting audiences to respond or locate themselves not only as ‘ethical consumers’ (if familiar to them) but also as ‘ecological citizens’ via the constellation of methods of performative engagement that the exhibition offered. Although situated on a busy high street in the centre of Bristol, our free exhibition offered nothing to buy nor contained information associated with ethical supply chain activities. The emphasis was not on local, fair-trade, organic or animal welfare–friendly products, nor was advice available about ‘how to food shop’: if we had done this, we would have been enacting the ethical consumer. Instead, we encouraged people to attend to different lived experiences with food preparation and eating. We facilitated experiences that challenged assumptions about the daily practice of food consumption, underlining our efforts to engage visitors in unexpected ways. For example, towards the end of the event, one individual entered the exhibition space looking to buy a gift for his father:

What was funny is he came in and [he saw] we were just doing baking and planting and this sort of stuff and I figured he’d be like, out the door. But he stood there; he was there for about half an hour I think if not longer, chatting about planting and talking about different ways . . . how do you grow this? Oh. How many melons would this get? You know? She’s [ELM volunteer] going, you know, it depends on where you put it, you know, so he had actually, he had a full, engaged discussion around food and plant, you know . . . but he was totally the kind of guy who wouldn’t have expected that, because he was so, like, you know, consumption man ready to just, you know, what can I buy?^50^

For this individual, the exhibition provided both a whimsical and unexpected high street experience and seeded different ideas about what can happen in ‘shops’ as he engaged with details about growing plants for food. Furthermore, he challenged our ideas about what the space could be used for and who might benefit or contribute to its unfolding. More significantly, the location of our exhibition within a pro-environmental festival as well as the dramatic green wall at the storefront produced a productive tension between varying conceptions of sustainability, resilience and social justice. The artist, Paul Hurley, reflects,

When visitors were coming in and out of the gallery, and I think maybe at first, because it was the [Festival of] Nature, and because of where it [was . . . ] [There are] a lot of kind of middle-class Bristol foodie people, you know, which I’m kind of partly one, I guess. But actually reading them to see how much food and ideals or opinions about food are kind of bound up with class prejudice and wealth and poverty and it just becomes so apparent when you’re looking at a basket of tinned baked beans and someone’s telling you why, where or what people should be eating organic, you know, and it’s that – this reality is so out of whack . . .^51^

Within Foodscapes, we found ourselves becoming more aware of the precariousness associated with food poverty and the uncanny presence of pro-environmental rhetoric within the setting. Passers-by who discussed buying local, buying fresh foods or buying organic foods seemed caught up in a set of food-related marketing slogans and being conspicuous consumers.^52^ It was notable how challenging it was to engage with foodstuffs in a manner other than through what one chose to buy. And yet through sustained engagement with the installation, conversations and practices were enacted that related to the skills of growing and baking – in other words – the experience of the installation enabled the foregrounding of ways to relate to food, other than as ethical consumers. However, so established are the practices of being an ethical food consumer, to follow the practices that can enact becoming an ecological citizen was not spontaneous but rather took time to come to the fore. Indeed, we recognised a striking contrast between the way some individuals described quite deliberate and varied food provisioning practices (e.g. locally or personally grown, healthy or organic options and so on) and many of our research participants who relied on the limited selection offered through food hand-outs for a significant part of their diet.

**Figure 4.**
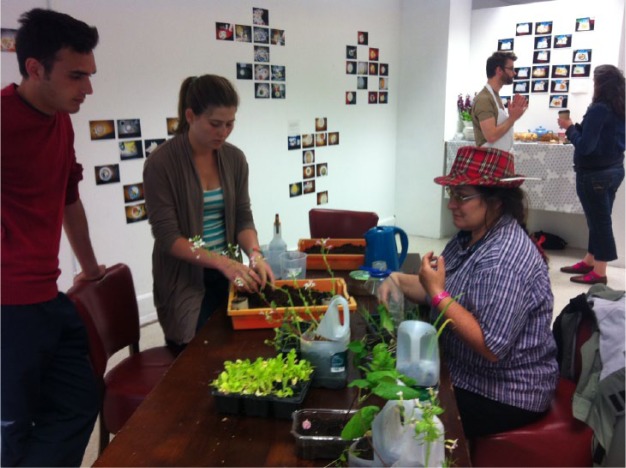
Planting exposition and workshop held during the exhibition.

The cultural arts and performance can work in particular ways to encourage political participation in novel ways around food. As Emma Roe writes in Goodman et al.,^53^ these practices can engage an audience through ‘mix[ing] the familiar with the strange and wacky, [they can] be effective at punctuating the everyday and in so doing become the stuff of memories, informing without didacticism’. One way to apprehend how the art produced space itself, to afford a juxtaposition of experiences between different people, materials and the processes and practices that entertain their enactment as ecological citizen, is through interpreting it through the writing of Brian Massumi. Juxtapositions, as Massumi would express it, are ‘the direct “pairedness” of pure, open contrast’,^54^ from which emerges ‘relating’. Massumi argues that it is from the contrasts that a figure of stability can emerge. This appears an interesting way to approach how the art-space engaged audience, for it uses the diverse experiences, skills and cultures that arrive in the space, affirmatively for the development of relating. And indeed our findings suggest that feelings and thoughts were generated through the juxtapositions of food experiences that the space offered, especially for those that lingered. Our claim that this experience enabled visitors to perform as ecological citizens is based on drawing people to relate to food ethics and food ecologies as something more than performing the role of an ethical consumer who can buy ethical products. Instead through focusing on shared food practices and shared food experiences – planting, eating, cooking, shopping, baking, digesting and preparing a meal – juxtapositions could emerge (see Figure 4). Notably, the human participants themselves were valued ingredients for creating juxtapositions, as anything non-human we had pre-assembled in the space, consequently there was a spontaneity and creativity that necessarily was embedded in the unplanned encounter between people, materials, the space and what could happen inside it.

### Implications for collaborative and participatory research practices

The experiences of people involved with the local food initiatives had affected us as researchers by engendering in us a responsibility to generate greater understanding of the predicament and politics of their plight. It strongly shaped how we did research:

Something that was really striking which we talked about during the process was how I guess our coming into it with that connection with the food bank clients, [. . . ] a sort of sense of protectiveness towards them, [. . . ] a kind of bond, the care, the duty, because [now] they were volunteering [to be involved in the project].^55^

We wondered how this affect on us could be mobilised to influence the experiences of the audience-participants through how the materialities of the space intra-acted with their previous food practices and histories. Here, following Latour, we were enacting ecological citizenship through how bodies or matter became ‘matters of concern’ through this intra-action.^56^ Interestingly, Puig’s use of the term ‘matters of care’^57^ that refers to a tendency for parts of the assemblage to be neglected is relevant here. For example, it is the intent of organisations such as TMTP to ensure that the recipients of emergency food aid are being kept on the radar and a ‘matter of care’ as opposed to one of neglect. This is where the political manoeuvre of ecological citizenship also instils an ethical impulse to act through matters of concern becoming matters of care. This resonates more closely with how we associated ourselves with the practices of TMTP, in wanting to actively work to support their goals. However, in the exhibition, this was achieved through purposeful design that sought to minimise didactic teaching methods and any sense of preaching, moralising or instilling a political will for social justice or detailing how to care. Speaking about how the space was composed, the artist recognised that

By toning down the profile, dominance of those organisations made for [a] more kind of open and liberated space. Occasionally people would think that Foodscapes was what we were trying to do, what we were trying to sell people. Are we trying to tell people about nutrition? Or are we trying to get people to shop locally? Or are we trying to, you know expecting that kind of organizational objective or [a] clear set of aims or things like that?^58^

This arrangement facilitated the coming together of diverse peoples, drawn to the space for its different and contrasting qualities. Furthermore, we suggest that these juxtapositions (of people and matter) contributed to more thoughtful and affective engagements with food practices and improved the quality of interactions. As one visitor reflected,

It made me think about the role of food in our society, our alienation from it, for the most part, for me anyway, I don’t have an allotment so all of my food comes from the store.^59^

Indeed, even without strongly worded propaganda about food inequalities and the ecologies of food production systems, this individual was able to discern important political and ecological messages and apply them to her personal food experience.

## Conclusion

Our approach encourages the enactment of becoming ecological citizen, in contrast to self-reflective approaches for encouraging political participation in agro-food politics, argued for by Morgan^60^ and that were the original framing of what it might be to become an ‘ecological citizen’ by Dobson.^61^ The two steps to becoming ecological citizenship outlined develop a politics through embodied practices that, first, foreground the activity of the human as operating with and in response to the matter of the world. And second, the arrangement of materials in space can work to instigate particular performances, for instance, by juxtaposing experiences and practices to engender affective responses and through situating political praxis within shared and distinctly different ways of making food connections. Throughout, we purposefully avoided didactic methods of exchange and relied on the juxtaposition of various food practices and materials to create non-linear experiences to solicit conversation and draw out meaning, since we felt that the juxtaposition of food materials and practices helped to bring people together from different backgrounds and disrupted taken-for-granted assumptions about consumption, food security and boundaries between art and the everyday. In this way, we have developed an approach for tackling some of the challenges of facilitating meaningful encounters between foods and people, for fostering care for others’ food insecurities and how they relate to the practices of becoming ecological citizen. Equally, we have contributed to debates on how food can be used to engage people to connect and care for others through food distribution and provisioning networks.
